# isomiR2Function: An Integrated Workflow for Identifying MicroRNA Variants in Plants

**DOI:** 10.3389/fpls.2017.00322

**Published:** 2017-03-21

**Authors:** Kun Yang, Gaurav Sablok, Guang Qiao, Qiong Nie, Xiaopeng Wen

**Affiliations:** ^1^Key Laboratory of Plant Resources Conservation and Germplasm Innovation in Mountainous Region – Ministry of Education, Institute of Agro-bioengineering, Guizhou UniversityGuiyang, China; ^2^College of Life Sciences, Guizhou UniversityGuiyang, China; ^3^Climate Change Cluster (C3), University of Technology SydneySydney, NSW, Australia

**Keywords:** miRNAs, isomiRs, functional profiling, PARE-seq, plants, post-transcriptional machinery

## Abstract

In plants, post transcriptional regulation by non-coding RNAs (ncRNAs), in particular miRNAs (19–24 nt) has been involved in modulating the transcriptional landscape in developmental, biotic and abiotic interactions. In past few years, considerable focus has been leveraged on delineating and deciphering the role of miRNAs and their canonical isomiRs in plants. However, proper classification and accurate prediction of plant isomiRs taking into account the relative features by which we define isomiRs, such as templated or non-templated is still lacking. In the present research, we present isomiR2Function, a standalone easily deployable tool that allows for the robust and high-throughput discovery of templated and non-templated isomiRs. Additionally, isomiR2Function allows for identification of differentially expressed isomiRs and in parallel target prediction based on both transcripts or PARE-Seq either using Targetfinder or Cleaveland. isomiR2Function allows for the functional enrichment of the detected targets using TopGO package. Benchmarking of isomiR2Function revealed highly accurate prediction and classification of isomiRs as compared to the previously developed isomiR prediction tools. Additionally, the downstream implementation of additional features allows isomiR2Function to be classified as a single standalone tool for isomiR profiling from discovery to functional roles. All in all, isomiR2Function allows the streamline processing of the miRNA-seq for the identification and characterization of isomiRs with minimal efforts. isomiR2Function can be accessed through: https://github.com/347033139/isomiR2Function.

## Introduction

Plants as a system are capable of regulating the stress and developmental responses either transcriptionally, or through post-transcriptional events. In view of emerging roles of non-coding RNAs (ncRNAs) such microRNAs (miRNAs) (Sablok et al., 2015; Karali et al., 2016), artificial microRNAs (Sablok et al., 2011), and circular RNAs (Sablok et al., 2016), it can be easily demonstrated that post-transcriptional regulation plays a critical role in understanding the elusive nature of the plant development and responses to varied kinds of environmental stresses. Post-transcriptionally, the role of the endogenous small RNAs has been well documented, described and widely demonstrated. High-throughput sequencing approaches have been widely applied to profile the role, diversity and functional elucidation of miRNAs in several model species such as *Arabidopsis thaliana*, *Brachypodium distachyon*, *Oryza sativa*, and crop species such as Sorghum and *Triticum* sp. (Guo et al., 2012; Yao and Sun, 2012; Bertolini et al., 2013; Li et al., 2013; Bologna and Voinnet, 2014). Interestingly, alongside the miRNAs, recently substantial focus has been leveraged to identify the variants of canonical miRNAs, henceforth termed as isomiRs, which have been recently shown to enhance miRNA predictions, binding efficiency (Guo et al., 2014) and also have been shown to be highly regulated as compared to the canonical miRNAs. One such example of isomiRs, are miRNA variants to canonical miR156, which have been shown to be widely regulated as compared to the canonical miRNAs (Baev et al., 2014). Taking into account increasing evidences of isomiRs, substantial efforts have been put forth to identify these miRNA sequence variants, which can be classified on the basis of the 5′ and 3′ nucleotide additions as well as substitutions, occurring as a result of the cleavage variations by ribonuclease Dicer-Like 1 (DCL) (Bartel, 2004). Although the biogenesis of isomiRs is still in naive, they have been further classified into three categories, i.e., 5′- isomiRs, 3′- isomiRs, and polymorphic isomiRs (Neilsen et al., 2012), and have been further grouped into templated (miRNAs length variants having homology to parent genes) and non-templated (non-templated nucleotide additions and/or post-transcriptional RNA edits resulting in no homology to parent gene) isomiRs based on occurrence of substitutions in isomiRs (Neilsen et al., 2012; Rogans and Rey, 2016).

In the past few years, considerable less efforts have been leveraged for the identification of isomiRs. It is worth to mention that in plants, no such algorithm yet exits which allows for the high-throughput simultaneous identification and functional classification of isomiRs. Previously developed algorithms for the identification of isomiRs such as isomiRID (de Oliveira et al., 2013), and sRNAtoolbox (Rueda et al., 2015), lack in providing the detailed profiling of the isomiRs, whilst taking into account the sequencing artifacts, expression based read support, visualization of the isomiRs with respect to read depth and mapping, target predictions and functional enrichment. Additionally, previously developed algorithms such as isomiRex (Sablok et al., 2013) are web-based and don’t provide classification and support system for analyzing wide array of plant species. Although the isomiR detection tool DeAnnoIso (Zhang et al., 2016) allows for the detection of isomiRs, however, it can be only accessible through the web interface and lacks supports to detect the isomiRs across wide range of plant species (only four plant species are supported). Another feature that the above stand-alone or the web-based algorithms lack is the integration of the PARE-Seq data and also the in-built support for the functional predictions and target classification. From the database point of view, isomiR database such as YM500 (Cheng et al., 2013) contains pre-analyzed isomiRs, with complete lack of support for the plant species.

Taking into account the relative lack of high-throughput detection of isomiRs, we developed isomiR2Function, which allows for the high-throughput detection of plant isomiRs from any miRNA-seq profiling study. isomiR2Function not only allows for the identification of the templated and non-templated 5′- isomiRs and 3′- isomiRs but also allows for the expression quantification using empirical bayes hierarchal model (EBSeq) and/or negative binomial distribution (DESeq). Prediction of biologically relevant target is an important criterion for the identification of isomiRs and their targets. isomiR2Function identifies target based on both alignment penalty using the Targetfinder as well as based on both alignment penalty and the appearance of decayed products using CleaveLand.

Following target prediction, it allows for functional enrichment of the identified targets using TopGO package. In parallel, isomiR2Function provides support for the visualization of read mapping on corresponding precursor sequences thus allowing for the identification and read based visualization of the detected isomiRs with respect to the precursor and canonical miRNAs. As compared to the previously published algorithms, isomiR2Function is a stand-alone tool that can be easily configured on any Linux and MAC operating system and can be used to profile isomiRs from any plant species ranging from dicot to monocot. To assess the robustness of the developed algorithm, we benchmarked isomiR2Function against the standalone isomiRID, which only profiles isomiRs and don’t provide support for the visualization, non-templated isomiR detection with classification and also doesn’t allow for target prediction and functional enrichment. To our knowledge, this is the first end-to-end streamlined workflow for the identification and classification of plant isomiRs, which allows for the identification and comprehensive classification of templated and non-templated isomiRs with support for read based visualization coupled with subsequent target prediction and functional enrichment.

## Materials and Methods

### Algorithm Implementation

isomiR2Function algorithm supports the parallel environment, and can be deployed on any Linux and MAC operating system. isomiR2Function has been developed on Ubuntu OS with 8 GB of RAM. The core algorithm of isomiR2Function is implemented in PERL version 5.014. For the identification of the differentially expressed isomiRs, we have implemented two different packages in R version 3.2: (1) DESeq (Anders and Huber, 2010) and (2) EBSeq (Leng et al., 2015), which have been integrated in the core algorithm of isomiR2Function. For the identification of the targets, two target prediction modules have been implemented, which provides support for transcript based target identification using TargetFinder (Fahlgren et al., 2007) and both transcript and degradome based PARE target identification using CleaveLand (Zhang et al., 2014). In-built functional enrichment support has been provided using TopGO version 2.24.0 (Alexa and Rahnenfuhrer, 2010) within the R framework 3.2.

### Sequence Datasets

To test the applicability of the isomiR2Function, we have profiled small RNAs datasets from three model species covering from dicot to monocot clade representing *A. thaliana* (SRR035251, SRR035252), *O. sativa* (SRR504362) and *B. distachyon* (SRR1033810). Additionally, for *A. thaliana*, degradome dataset (SRR1039524) was also included for target predictions (see Supplementary File 1 for details). For comparative analysis, isomiR2Function was benchmarked against isomiRID (de Oliveira et al., 2013) (see Supplementary File 1 for details).

## Results and Discussion

### isomiR Detection (Templated and Non-templated)

Recently, several studies have widely elucidated the role of the isomiRs (canonical miRNAs variants) in physiological responses and also demonstrated that the canonical variants are able to affect the miRNA stability and selection (Guo et al., 2014), especially the 5′- isomiRs, which might have different seed region as compared to the canonical miRNAs. It is worth to mention that in case of humans, cooperative ability of the miRNAs and isomiRs has been shown to be a way to increase the microRNAome targeting common biological pathways (Cloonan et al., 2011). Although these canonical variants have been defined as ‘templated’ and ‘non-templated’, based on the subsequent processing of DROSHA/DICER enzymatic machinery (Zhang et al., 2016), in-depth profiling of these 5′- isomiRs and 3′- isomiRs is still evolving in plants small RNA machinery. To provide supports for the high-throughput detection of the templated and non-templated isomiRs in plants, we have developed isomiR2Function, which executes in parallel as well as single thread environment.

**FIGURE 1:**
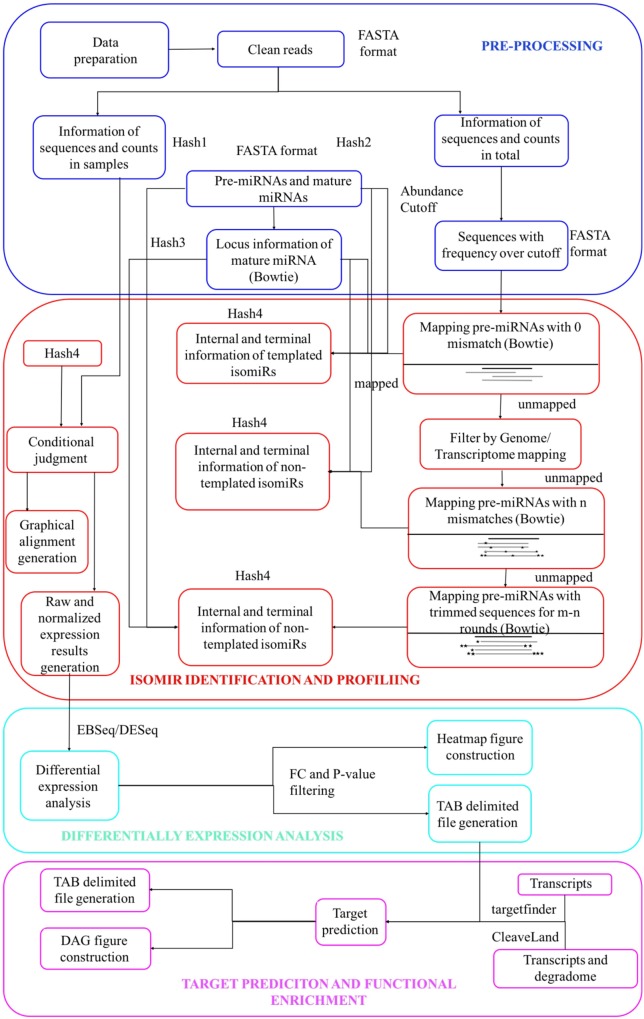
**Schematic view of the isomiR2Function workflow**.

**FIGURE 2:**
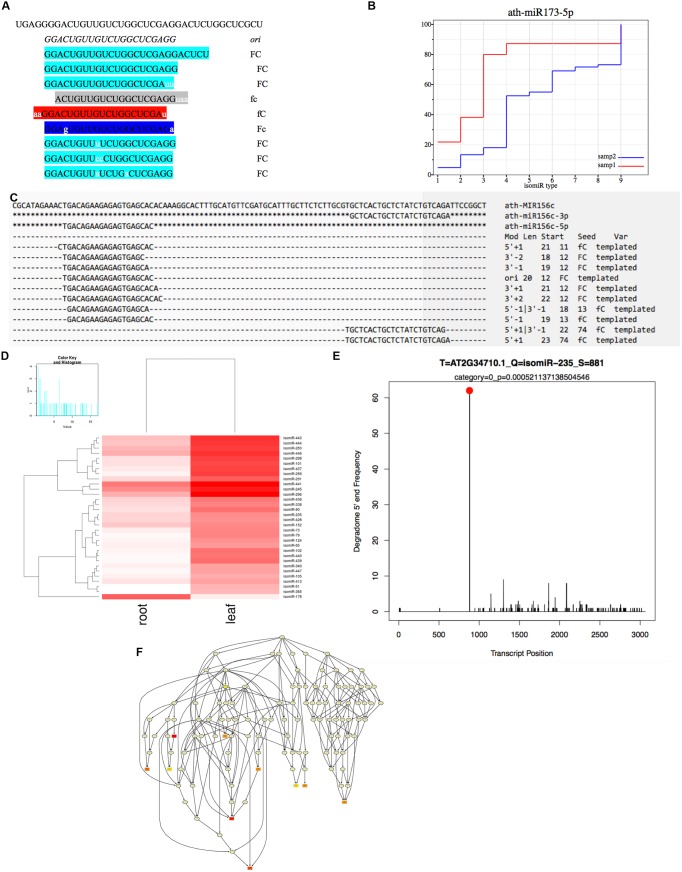
**Integrated workflow of the isomiR detection and functional analysis in isomiR2Function. (A)** Shows the seed corrupt algorithm; **(B)** Displays the KS plots for canonical miRNAs with more than one detected isomiRs. The *x*-axis indicates different types of isomiRs, the *y*-axis indicates the abundance ratio of corresponding isomiR to the total isomiRs; **(C)** Shows the alignment view of isomiR detection, where the top sequence is the pre-miRNA followed by the canonical miRNA. Non-templated bases are indicated with lower case. Mod suggests the terminal drift of isomiRs; **(D)** Shows the differential expression analysis module using DESeq and EBSeq or intersection of the two algorithms; **(E)** isomiR2Function supports target prediction using CleaveLand for the PARE and transcript data both or Targetfinder for transcript data only and **(F)** shows the functional enrichment module of the predicted targets using the TopGO package.

**Figures 1**, **2** describe the workflow of isomiR2Function, which consists of several modules from the pre-processing of the miRNA-seq reads to the end visualization of the detected isomiRs with read based support and also functional enrichment of the predicted targets using either transcript based or PARE-Seq based approaches. Pre-processing and isomiR identification modules of isomiR2Function call pre-processing of the sequenced small RNAs library by adapter cleaning using the local alignment of the defined 5′ and 3′- adapters and uses cutadapt as an adaptor cleaning tool (Martin, 2011). Adapter cleaned and collapsed reads are mapped to RFAM^1^, tRNAs^2^, and plant snoRNAs database^3^ to filter the reads originating from other ncRNAs. The adapter cleaned, filtered and collapsed small RNAs were then mapped to pre-miRNAs allowing no mismatch using bowtie (Langmead et al., 2009). isomiR2Function define templated isomiRs by comparing the mapping information of the small RNAs tags and canonical miRNAs on pre-miRNAs.

For non-templated isomiR detection, in case of sequences potentially originated from multiple pre-miRNAs, only best hits will be analyzed to avoid mapping ambiguity. Furthermore, unmapped sequences will be mapped to genome or transcriptome to decrease false positive rates of non-templated isomiRs identification by filtering mapped sequences. Lastly, sequences, which does not show mapping on any region of genome are re-mapped on pre-miRNA allowing ‘n’ mismatches, where ‘n’ is defined by parameter ‘s’. Taking into account above features, for non-templated isomiR detection, isomiR2Function executes in two steps: (1) In the first step, adapter cleaned tags will be mapped on pre-miRNAs with user-defined ‘n’ mismatches thus allowing the internal SNPs flexibility. Since the distribution of the SNPs can be random or tandem, isomiR2Function further checks the positions of the allowed mismatches. If the allowed mismatches are present at 3′- or 5′- end then the detected isomiRs will be classified under the category of the terminal substituted ones whereas if the allowed mismatches do not contribute to the terminal substitutions then isomiR2Function classifies them as MS for having internal random SNPs, CV for having both internal SNPs and terminal substitutions and TS for having internal tandem SNPs. Since the non-templated isomiRs are mostly by-products of the nucleotidyl transferases possessing 5′–3′ uridyltransferase and/or adenyltransferase activity, implementation of the aforementioned will allow the simultaneous detection of the non-templated isomiRs with internal SNPs and/or with terminal substitutions. (2) For the unmapped tags, detected in the first step, isomiR2Function will execute the additional recursive trimming and mapping step. In case of the recursive mapping and trimming, adapter cleaned tags, which do not map to the pre-miRNAs with the user defined ‘n’ mismatches, one nucleotide will be trimmed from either 5′ end or 3′ end at each recursive cycle and then will be re-mapped on pre-miRNAs for ‘m–n’ round, where ‘m’ is defined by parameter ‘r’. This recursive trimming and mapping continues for each of the adapter cleaned unmapped tags.

### isomiR Differential Expression, Target Prediction, Functional Enrichment, and Visualization

For detected isomiRs (templated and non-templated), isomiR2Function provides support for the differential expression analysis either using the DESeq (Anders and Huber, 2010) or EBSeq (Leng et al., 2015). Taking into account the replicate and non-replicate small RNAs sequencing datasets, we implemented both DESeq, EBseq or both (intersection of the top isomiRs taking into account both the algorithms), which allows for the identification of the differentially expressed isomiRs across non-replicate and replicate small RNAs datasets. Following the detection of the differentially expressed isomiRs, isomiR2Function will automatically plot the top 50 differentially expressed isomiRs and will also provide a TAB delimited file of all differentially expressed isomiRs for further exploratory analysis. Following expression analysis, differentially expressed isomiRs will be passed for the transcripts based target identification using TargetFinder (Fahlgren et al., 2007) or degradome based PARE target identification using Cleaveland (Zhang et al., 2014) as defined by the user and the data availability. In-built support for the functional enrichment using TopGO (Alexa and Rahnenfuhrer, 2010) allows for the functional enrichment of the identified targets for hypothesis validations. At the end of each analysis run, isomiR2Function provides a summary file describing several informative descriptions such as total number of isomiRs, canonical and non-canonical isomiRs, complex isomiRs (isomiRs having more than one pre-miRNAs), most abundant isomiR pre-miRNA, and a summary of the 5′ end and 3′ modifications after successful completion of the isomiR profiling (**Table 1**).

**Table 1:** Statistics of templated isomiRs detection in *Arabidopsis thaliana*, *Brachypodium distachyon*, and *Oryza sativa*.

Type of isomiRs	*A. thaliana*	*B. distachyon*	*O. sativa*
Total isomiRs	120	290	375
Complex isomiRs	55	74	125
Canonical isomiRs	61	118	125
Non-canonical isomiRs	4	98	125
Pre-miRNAs generating isomiRs	58	103	150
Most abundant isomiR pre-miRNA	ath-MIR166a	bdi-MIR444b	osa-MIR7695
Having 5′- addition only	9	6	3
Having 3′- addition only	15	17	9
Having 5′- deletion only	3	14	25
Having 3 prime deletion only	18	28	26
Having 5′- addition and 3′- addition both	1	0	2
Having 5′- addition and 3′- deletion both	15	23	30
Having 5′- deletion and 3′- addition both	0	28	25
Having 5′- deletion and 3′- deletion both	0	2	5

### Benchmarking isomiR2Function

**FIGURE 3:**
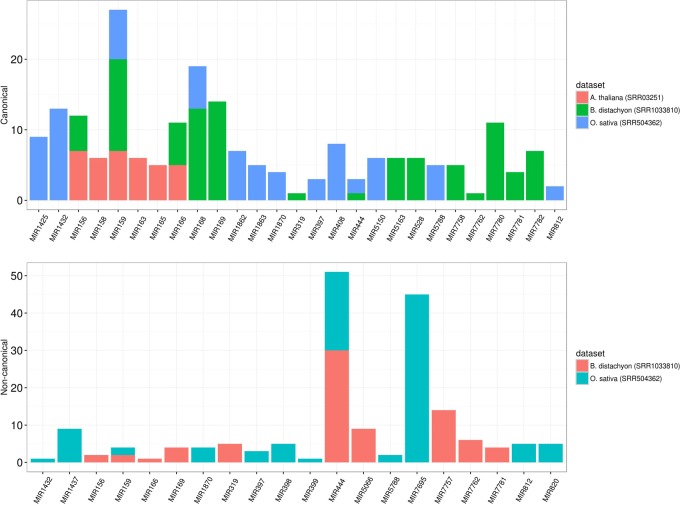
**Different preference of isomiRs across monocot to dicot**. The *y*-axis indicates the number of isomiR types and the x-axis indicates different families from monocot (*Brachypodium distachyon* and *Oryza sativa*) and dicot (*Arabidopsis thaliana*). In dicot, the most abundant isomiRs were canonical isomiRs whereas the most abundant isomiRs were non-canonical isomiRs in monocots. MIR444 generates a considerable number of non-canonical isomiRs in both *B. sistachyon* and *O. sativa*, although the most abundant family in *O. sativa* is MIR7695.

To assess the robustness of isomiR2Function, we benchmarked the developed algorithm across monocot and dicot small RNAs datasets to access the diversity and class of the identified isomiRs. isomiR2Function revealed a wide diversity of the identified isomiRs (**Table 1** and **Figure 3**) revealing 3′- additions/deletions as the most abundant event for isomiR variations, which is in line with the previous reports (Rogans and Rey, 2016). To assess the sensitivity and specificity of the algorithm, we compared isomiR2Function and isomiRID to access the accuracy of the implemented algorithm with settings: (1) at most 1 internal SNPs and 6 terminal substitution. (2) from 18 to 26 nt in length. (3) support for the identified isomiRs with only one reads from the sequenced data. (4) at least 16 nt of overlap of the defined canonical isomiRs sequences with canonical miRNAs. Benchmarking analysis revealed high accuracy of isomiR predictions by isomiR2Function as compared to previously published isomiRID in terms of classifying the the non-canonical isomiRs and non-templated canonical isomiRs (**Table 2** and Supplementary File 1). Additionally, we observed that isomiRID takes into account the length for recursive trimming and mapping of the trimmed tags as compared to the original adapter cleaned tags. Lastly, isomiRID reported isomiRs with several terminal substitution and with length beyond the user-defined length range thus resulting in incorrect graphical alignments of the identified isomiRs (Supplementary File 1). As compared to isomiRID, isomiR2Function reported correctly previously defiend isomiRs, which were predicted by isomiRID (**Table 2**) plus predicted additional specific non-templated isomiRs, which were not reported by isomiRID. Comparative results for dataset SRR035251 and SRR035252 can be browsed at Supplementary File 1 and https://drive.google.com/file/d/0B-w2z1YjW6TieG9jTUxhSmNwTTQ/view.

**Table 2:** Comparative assessment of isomiR profiling using isomiR2Function and isomiRID.

	isomiR2Function						isomiRID					
	Templated			Non-templated			Templated			Non-templated		
	ath	bdi	osa	ath	bdi	osa	ath	bdi	osa	ath	bdi	osa
Total	383	1613	2734	3959	8881	10946	383	1613	2734	3905	6869	10946
Complex	120	230	541	2299	2471	3836	120	230	541	2284	2270	3836
Canonical	200	629	836	1504	3748	2921	200	629	836	1499	3108	2921
Non-canonica	63	754	1357	156	2662	4189	63	754	1357	122	1491	4189
5a	14	28	33	250	133	82	14	28	33	250	111	82
3a	41	42	61	392	491	299	41	42	61	391	474	299
5d	17	53	89	29	440	189	17	53	89	29	411	189
3d	47	98	132	149	553	323	47	98	132	148	520	323
5a3a	6	9	18	69	39	30	6	9	18	67	22	30
5a3d	50	164	219	212	461	442	50	164	219	212	242	442
5d3a	17	210	224	44	511	549	17	210	224	44	289	549
5d3d	8	25	60	8	97	67	8	25	60	7	62	67
No drift	0	0	0	351	1023	940	0	0	0	351	977	940
Both	383	1613	2734	3905	6869	7877	383	1613	2734	3905	6869	7877
Specific	0	0	0	54	2012	3069	0	0	0	0	0	0

***^∗^**ath = *Arabidopsis thaliana*; bdi = *Brachypodium distachyon*; osa = *Oryza sativa*; Complex, isomiRs with multiple origin; Canonical, isomiRs have enough bases overlapped with canonical miRNAs; Non-canonical, isomiRs do not have enough bases overlapped with canonical miRNAs; 5a = 5′ terminal addition; 3a = 3′ terminal addition; 5d = 5′ terminal deletion; 3d = 3′ terminal deletion; 5a3a = 5′ terminal addition and 3′ terminal addition; 5a3d = 5′ terminal addition and 3′ terminal deletion; 5d3a = 5′ terminal deletion and 3′ terminal addition; 5d3d = 5′ terminal deletion and 3′ terminal deletion; No drift, isomiRs identified at the same place of canonical miRNAs on the pre-miRNAs; Both, identified by both tools; Specific: Identified specifically. isomiRID available from https://github.com/lfelipedeoliveira/isomiRID*

## Conclusion

To conclude, isomiR2Function allows for streamline detection of templated and non-templated isomiRs with following additional functionalities: (1) It allows for customizable length range, seed corrupt tolerance and minimum sequencing depth for isomiR identification; (2) Identification of isomiRs on pre-miRNA not overlapping with canonical miRNAs; (3) Information on isomiRs biogenesis using Ks plots; (4) Support for differential expression analysis of the identified isomiRs using DESeq (Anders and Huber, 2010) and EBSeq (Leng et al., 2015); (5) Detailed classification of multiple internal substitution. isomiR2Function classifies isomiRs with multiple internal substitution in more detail, i.e., MS for having internal random SNPs, CV for having both internal SNPs and terminal substitutions, TS for having internal tandem SNPs, 5V/3V for 5′/3′ end substitutions; (6) Support for transcript based target identification using TargetFinder (Fahlgren et al., 2007) or degradome based PARE target identification using Cleaveland (Zhang et al., 2014); (7) Functional enrichment of isomiRs targets for biological implications. Taking into account the implemented functionalities, we believe that isomiR2Function will facilitate the large-scale discovery of isomiRs across the plant species to gain and strengthen the role of the miRNA variants in post-transcriptional regulatory events.

## Author Contributions

KY performed the coding for the isomiR identification. GS implemented target predictions, differential expression analysis, GO enrichment and guided the designing of the isomiR2Function algorithm. GQ and QN prepared the datasets for validation of the isomiR2Function. KY, GS, and XW wrote the manuscript. XW originated the concept.

## Conflict of Interest Statement

The authors declare that the research was conducted in the absence of any commercial or financial relationships that could be construed as a potential conflict of interest.

## Footnotes

1 rfam.xfam.org/

2 rfam.xfam.org/

3 http://bioinf.scri.sari.ac.uk/cgi-bin/plant_snorna/home
