# Supplementary material for: isomiR2Function: An Integrated Workflow for Identifying MicroRNA Variants in Plants
Source: Front Plant Sci. 2017 Mar 21;8:322. doi: 10.3389/fpls.2017.00322 (PMC5359237; doi:10.3389/fpls.2017.00322)
Supplement: Supplementary file 1 [file Presentation_1.pdf]

isomiR2Function Quick Start

(v2017.02.14)

isomiR2Function integrates analyses from identification to functional analysis of isomiRs. This tool uses its original algorithm for isomiR identification and classification, and calls other widely used tools for downstream analysis.

DOWNLOAD

isomiRFinder is available at:

https://github.com/347033139/isomiR2Function

CONTENT

This package has six original directories:

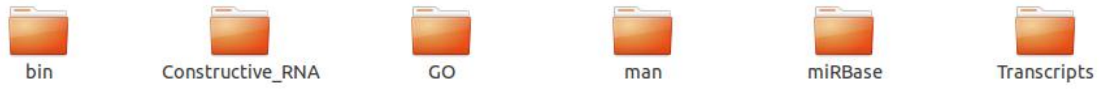

The first one contained the whole potential scripts to be used. Descriptions of new changes for isomiR2Function will be added into the forth directory by version. The fifth directory stores the sequences of all the canonical miRNAs and their hairpins deposited in the latest miRBase (release 21 now). The other three are initially empty.

SCRIPTS IN BIN

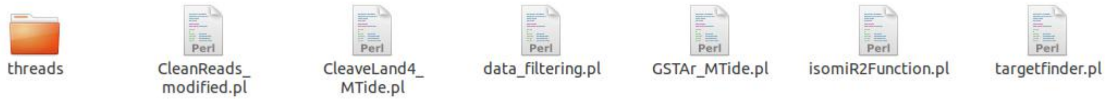

The bin directory has above one sub-directory and six Perl scripts. ‘CleaveLand4.pl’ and ‘targetfinder.pl’ is called by isomiR2Function for target prediction. ‘CleaveLand4.pl’ is the modified version of CleaveLand pipeline from MTide tool package, which is able to detect the cleavage ability at 9-11 nt of isomiRs from 5’-3’ orientation. ‘CleanReads_modified.pl’ and ‘data_filtering.pl’ is for inputting data preparation. The first one does quality control, adapter trimming and sequence collapsing. FASTA/FASTQ is supported as input. The second one filters out

unexpected small RNAs from data, i.e. rRNAs and tRNAs. If no unexpected sequences are provided, 'data_filtering.pl' will search directory 'Constructive_RNA' for a file named 'constructive_RNA.fa' to filter out unwelcome sRNAs. 'GSTAr.pl' is the alignment tool for 'CleaveLand.pl'. 'isomiR2Function.pl' is the main script of our package. In threads, you will find scripts named CleaveLand4_MTide.pl, GSTAr_MTide.pl and isomiR2Function.pl which allows identify isomiRs with multiple threads.

DEPENDENCY

Perl version: 5.014 or above;

Perl models: Getopt::Std for CleaveLand4_MTide.pl, data_filtering, GSTAr_MTide.pl, isomiR2Function.pl and targetfinder.pl;
Getopt::Long for CleanReads_modified.pl;
Math::CDF for CleaveLand4_MTide.pl;
File::Basename for CleanReads_modified.pl and isomiR2Function.pl;
File::Spec for data_filtering.pl and isomiR2Function.pl;
Thread::Semaphore for GSTAr_MTide.pl;
threads for GSTAr_MTide.pl;
SVG for isomiR2Function.pl;
List::Util for isomiR2Function.pl;
File::Temp targetfinder.pl;
constant for targetfinder.pl.

R version: 3.22 or above;

R package: DESeq;
EBSeq;
topGO;
gplots.

Python: 2.7 or above.

Tools: bowtie, bowtie-build and cutadapt.

!!!IMPORTANT replace the isomiR2Function.pl, CleaveLand4_MTide.pl and

GSTAr_MTide.pl with files having the same name in 'threads' directory if your perl is configured wilt '-Dusethreads'. Which will make isomiR2Function.pl work much more efficiently.

ONLINE RESOURCES

To make convenience for users, a list of 21 widely researched species is uploaded online. Their GO backgrounds are stored in the corresponding directories. The list will be updated if we have made online data for a new species. All these data were generated from www.phytozome.net. For reads filtering, we have prepared plant specific tRNA, rRNA and snoRNA sequences. These files are deposited into the directory 'Constructive_RNA' of online version of isomiR2Function. tRNAs and rRNAs are from NCBI Nucleotide database (http://www.ncbi.nlm.nih.gov/nuccore) and plant specific snoRNAs are from Plant snoRNA Database (http://bioinf.scri.sari.ac.uk/cgi-bin/plant_snorna/home). A template of config_file is at the root path of isomiR2Function project too. In that file, we provides information of three datasets from two different tissues for isomiRs identification and differential expression analysis.

FILE PREPARING

For a thorough analysis workflow of isomiR2Function, the user should prepare eight files before running isomiR2Funtion.pl: 1) clean_reads.fa (-t). This file will contains all the small RNAs which will be identified as isomiRs. All the reads will be unique and the headers should be in a format as ">label_rank_xcount". To avoid any potential problem during the downstream analysis, we strongly recommend the user to use 'CleanReads_modified.pl' for adapter trimming, quality control and converting FASTQ data to FASTA. 'CleanReads_modified.pl' calls python package cutadapt for trimming. You should add cutadapt in your PATH environment variable or revise the path to call cutadapt in the script. 2) pre-miRNA.fa (-p) and canonical-miRNA (-a). These two files will contain the sequences of species specific pre-miRNAs and canonical miRNAs. The header of 'pre-miRNA.fa' and 'canonical-miRNA.fa' should

be the same except '-3p' or '-5p' is appended after the name of mature miRNAs, e.g. '>ath-miR156a-5p' for mature miRNA and '>ath-miR156a' for precursor. Case of letter is not sensitive here. If the subject species is covered by miRBase, you can use the parameter '-e' instead of providing above two files or you can use '-p', '-a' and '-e' at the same time to run isomiR2Function with your newly identified miRNAs along with those deposited in miRBase. 3) genome_index_name. This name of path will direct isomiR2Function to the path of genome indexes. You can generated these files by bowtie-build. If the genome of your subject is not accessible, transcriptome can be used instead. 4) config_file.tbl. This file will contains the details of the dataset you use for analysis, and isomiR2Function will read this file to understand your experiment design better. We have uploaded a template of this file online (see ONLINE RESOURCES). 5) GO_background.tbl. This file will contains transcript sequences of your subject for target prediction. One geneID a line, and their GO terms are appended after delimited by a TAB. Different GO terms are separated by commas. The geneIDs of this file should be consistent with that in 'transcript.fa'. You can prepare this file from www.phytozome.net. 6) PARE.fa. This file will be needed only if the user call the parameter '-i C'. It contains the degraded sequences of mRNA and the user should not remove the redundancy. 7) transcripts.fa. This file will contain the transcripts of your subject. You can prepare this file from www.phytozome.net.

RUNNING

To start running the isomiR2Function, easily execute the command line like:

```
User:~$ isomiR2Function.pl -t clean_reads.fa [-p pre-miRNA.fa -a canonical-miRNA.fa | -e ORG] -g genome_index -y config_file.tbl [options]
```

Where options are changeable parameters of isomiRFinder. isomiRFinder calls bowtie for mapping, thus you should put 'bowtie-build' and 'bowtie' in PATH environment value.

OPTIONS

-t	<file>	Unique small RNA sequence files separated by comma, fasta format, forced
-p	<file>	Pre-miRNA sequence file, fasta format, come with -m
-a	<file>	miRNA sequence file, fasta format, come with -p
-g	<file>	Genome or transcriptome index built by bowtie-build, fasta format, forced
-y	<file>	Dataset config file, TAB delimited, forced
-w	<file>	GO background file, one gene a line, forced
-j	<file>	Degradome sequencing data, fasta format, come with '-i C', -m, -q and -u
-n	<file>	Transcripts sequences, fasta format, forced
-o	<dir>	The directory where you want to save the results.
-e	<str>	Species of subject, miRBase taxon reference
-s	<int>	Max internal snp allowed (0)
-r	<int>	Max terminal substitution allowed, >= -s (0)
-f	<int>	Min isomiR abundance (0)
-d	<int>	Min overlapping with canonical miRNA (16)
-L	<int>	Min isomiR sequence length (16)
-l	<int>	Max isomiR sequence length (28)
-c	<int>	Max corrupt in seed region (0)
-S	<int>	Start of seed region on original miRNA (2)
-E	<int>	End of seed region on original miRNA (8)
-F	<int>	Log2 foldchange cutoff of differential expression analysis (1)
-P	<int>	P value cutoff of differential expression analysis (0.05), used for DESeq only if have replicates
-b	<str>	Calling which algorithm for differential expression analysis, DESeq or EBSeq (EBSeq)
-i	<str>	Calling which pipeline for target prediciton, C (CleaveLand) or T (targetfinder,default)
-m	<str>	P value cutoff of target prediction when calling CleaveLand (0.05), come with '-i C', -j, -q and -u
-q	<int>	How many mismatch will be allowed when mapping degradome data on

		transcripts (0), come with '-i C', -m, -j and -u
-u	<int>	Which level of categories should be allowed, 0 to 4 (4), come with '-i C', -m, -j and -q
-k	<int>	Alignment score cutoff of target prediction when calling targetfinder (4)
-T	<int>	Number of cores for parallel analysis (1)
-v		Keep tmp files remain for debug
-h		Call out the manual of isomiR2Function

WORKFLOW

The isomiR2Function workflow has seven main steps:

Step 1, sequence abundance in each libraries and in total are stored in Hash1 and Hash2, respectively. Simultaneously, canonical miRNAs are mapped on pre-miRNAs using bowtie and their locus information is stored in Hash3. Abundance filtering is carried out here to avoid unnecessary mapping to speed up process.

Step 2, sequences passed the filtering are mapped on pre-miRNAs using bowtie. Internal and terminal information of these sequences generated by compared to canonical miRNAs is checked. Sequences with allowed 5' and 3' coordinates and seed corruptness are stored in Hash4 as templated isomiRs. The file of unmapped sequences is created by bowtie and used for subsequent analysis.

Step 3, unmapped sequences from above step are filtered with genome or transcriptome sequences by mapping against to decrease false positive results. Then, sequences failed in mapping to other region of the reference are mapped on pre-miRNAs allowing 'n' mismatches, where 'n' is defined by parameter '-s'. Mapping results were fully analyzed by core algorithm of isomiR2Function. Terminal variations, internal substitution were then sort out from mixed type, separately. Internal and terminal information as well as seed corruptness of passed sequences is appended to Hash4. For sequences who might originate from multiple pre-miRNAs, only best hits were analyzed because the fewest variation these hits get.

Step 4, Sequences do not hit the pre-miRNAs from above step are trimmed from their 5' or 3' terminal followed by mapping them on pre-miRNAs again allowing no

mismatch. The trimming and mapping events are carried on for ‘m-n’ round, where ‘n’ is defined by parameter ‘-r’. Internal and terminal information as well as seed corrupt of the original sequences, who hit the pre-miRNAs after trimming, are appended to Hash4.

Step 5, Hash4 is passed to core algorithm of isomiR2Function. Graphical alignment file is generated and the details of this isomiR are appended after. Sequentially, conditional judgment is carried out, quantification file for isomiRs in different samples are built.

Step 6, EBSeq or DESeq is called by isomiR2Function for differential expression analysis of isomiRs. TAB delimited quantification file is written. Heatmap graph is also constructed for visualization.

Step 7, Targetfinder or CleaveLand pipeline is called by isomiR2Fucntion for target prediction followed by the GO enrichment of these predicted targets of differentially expressed isomiRs. Finally, significantly enriched GO terms are saved in a TAB delimited result file and a DAG figure consists of those GO terms is drawn.

OUTPUT

Templated and non-templated isomiRs are detected and analyzed separately by isomiR2Function. Normally, isomiR2Funstion will generated two sub-directory in the root path of outputs:

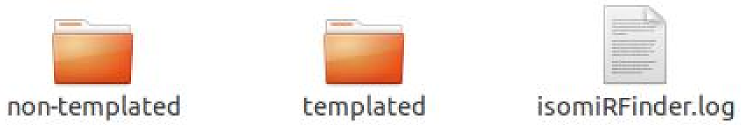

The lasst one stores the details of parameter setting of running. Both directories will have results showing bellow.

The result files of isomiR2Function can be mainly divided into five part:

1 isomiR identification

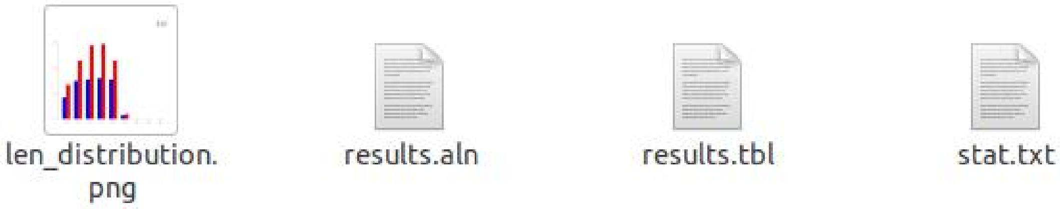

From the left to right are the results of the original algorithm of isomiR2Function. The first one shows the length distribution of all isomiRs between different samples. The second one is the graphical alignments of isomiRs to corresponding pre-miRNAs.

```
:TCTTTCTGTCAGATTCCGGTGCTGATCTCTTT    ath-MIR156a
:TCTTTCTGTCAGA*******************    ath-miR156a-3p
*********************************    ath-miR156a-5p
---------------------------------    Mod       Len      Start    Seed     Var
---------------------------------    5'+4|3'-1          23       17       fC       5V: TAT
---------------------------------    5'+3      23       18       fC       SS: 1:A>T
---------------------------------    5'+2|3'-2          20       19       fC       SS: 1:C>A
---------------------------------    5'+2      22       19       fC       SS: 1:C>A
---------------------------------    5'+2      22       19       fC       5V: 0:A>T
---------------------------------    5'+1|3'-1          20       20       fC       3V: AC
---------------------------------    5'+1|3'-1          20       20       fC       SS: 16:A>C
---------------------------------    5'+1|3'-1          20       20       fC       5V: 0:C>A
---------------------------------    5'+1|3'-1          20       20       fC       3V: 19:A>G
---------------------------------    5'+1      21       20       fC       SS: 18:C>A
---------------------------------    5'+1      21       20       fC       SS: 19:A>T
```

Above is an example of result.aln. The top sequence is the pre-miRNA followed by the canonical miRNA under it. The below aligned sequences are identified isomiRs. Non-templated bases are indicated with lower case. Mod suggests the terminal drift of isomiRs. For example, '3'+1;5'-2' means this isomiR has one nucleotide addition at 5' terminal. and two nucleotide deletion at its 3' terminal. Len suggests the length of isomiRs. Start suggests the relative start of isomiRs on pre-miRNAs. Seed suggests the corruptness happening in seed region. Var suggests the base variation of isomiRs. SS means single SNP, MS means multi-SNP, CV means compounded variation, TS means tandem SNP. For example, SS: 16:A>C means single SNP is detected at the 16th relative site on isomiR converting A to G. The seed corruptness is defined as the following graph.

```
UGAGGGGACUGUUGUCUGGCUCGAGGACUCUGGCUCGCU
     GGACUGUUGUCUGGCUCGAGG                    ori
     GGACUGUUGUCUGGCUCGAGGACUCU               FC
     GGACUGUUGUCUGGCUCGAGG                    FC
     GGACUGUUGUCUGGCUCGAuu                    FC
       ACUGUUGUCUGGCUCGAGGuaa                 fc
   aaGGACUGUUGUCUGGCUCGAu                     fC
     GGAgUGUUGUCUGGCUCGAGa                    Fc
     GGACUGUUuUCUGGCUCGAGG                    FC
     GGACUGUUccCUGGCUCGAGG                    FC
     GGACUGUUaUCUGaCUCGAGG                    FC
```

The third one is a TAB delimited table file for all identified isomiRs. Information about sequence, mismatch, base variation, 5' and 3' drift, sample information, and RPM are stored in this file.

The last one calculates statistic information of identified isomiRs.

```
Number of total isomiRs: 241
Nunmber of complex isomiRs: 92
Number of canonical isomIRs: 117
Number of non-canonical isomIRs: 32
Number of pre-miRNAs generating isomiRs: 113
Most abundant isomiR pre-miRNA: ath-MIR161
Number of 5 prime addition: 18
Number of 3 prime addition: 20
Number of 5 prime deletion: 15
Number of 3 prime deletion: 33
Number of 5 prime addition and 3 prime addition: 1
Number of 5 prime addition and 3 prime deletion: 25
Number of 5 prime deletion and 3 prime addition: 3
Number of 5 prime deletion and 3 prime deletion: 2
```

2 miRNA biogenesis and processing analysis

KS-test plots are built for canonical miRNAs with more than one detected isomiRs. The results are stored in 'ks_test' directory. The x axis indicates different types of

isomiRs, the y axis indicates the abundance proportion of corresponding isomiR to the total isomiRs.

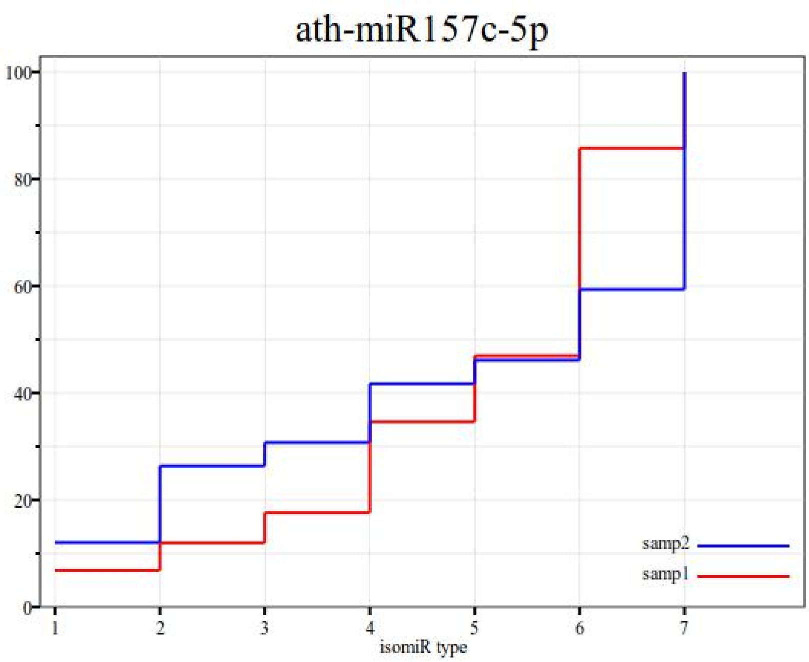

3 Differential expression analysis

isomiR2Function supports two algorithm for differential expression analysis, DESeq and EBSeq. Both algorithm will result three file in 'DE' directory.

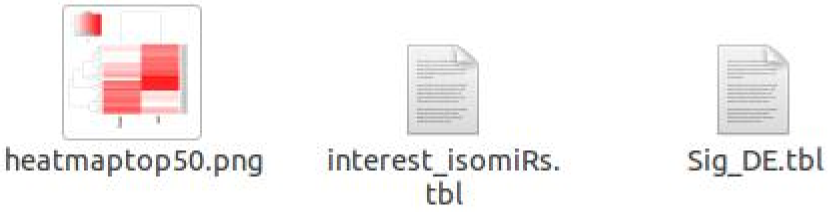

The first one is a heatmap graph of top 50 differentially expressed isomiRs. The third one contains the details of isomiRs whose fold changes and p values are within the cutoff. The second one is a TAB delimited table file which contains the names and sequences of differentially expressed isomiRs. If the experiment has replicates. The intersection between DESeq and EBSeq results can be investigated using parameter '-b both'. This analysis is allowed only if experiment has replicates. If not, the isomiR2Function will complain and exit. The results of DESeq depends on whether experiment has replicates too. If does, the differentially expressed isomiRs will be

selected by both p value and fold change, if doesn't, then the differentially expressed isomiRs will be selected by only fold change.

4 Target prediction

isomiR2Function supports two pipeline for target prediction of isomiRs, CleaveLand and targetfinder. Both pipeline will result a TAB delimited result file in 'targets' directory and a file listing the targets of differentially expressed isomiRs in 'topGO' directory, named 'gene_of_interest.txt'. If user calls CleaveLand pipeline for target prediction. Then an additional sub-directory 'Tplots' will be built in 'targets' directory. All the T-plots of identified targets will be drawn in this directory.

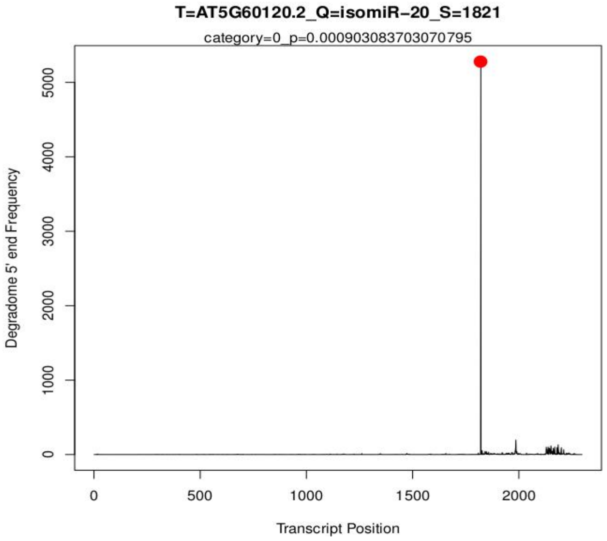

Above is an example of CleaveLand-MTide generated T-plot. x axis indicates the positions on transcripts, y axis indicates the number of identified cleavage happened. Red spot indicates the cleavage result of identified isomiR::transcript pair.

5 GO enrichment

R package 'topGO' is called by isomiR2Function for GO enrichment of targets of differentially expressed isomiRs. The p value is calculated by Fisher's exact test and the cutoff is set at 0.001. TAB delimited result files and DAG graphs for significantly enriched GO terms based on Biological Process, Molecular Function and Cellular Component will be constructed in 'topGO' directory.

VALIDATION

The practicability of isomiR2Function has been validated by running with two small RNA sequencing (SRR035251 and SRR035252) and one PARE sequencing data (SRR1039524). The inputs, outputs and command line to run the validation can be accessed at: https://drive.google.com/file/d/0B-w2z1YjW6TiRVJ5QTFidGI5MUE/view?usp=sharing and checked from online file 'validation.tar.bz2'. Canonical and pre-miRNAs were downloaded from miRBase (release 21). Genome and transcript sequences are downloaded from www.phytozome.net.

To estimate the sensitivity and accuracy of isomiR2Function, we compared the behave of isomiR2Function against a previously published tool isomiRID using datasets from *Arabidopsis thaliana* (SRR035251), *Oryza sativa* (SRR1033810) and *Brachypodium distachyon* (SRR504362). Tools are run allowing the following parameters: 1 At most one internal mismatch against pre-miRNA is allowed for isomiR identification. 2 At most four bases of truncation and editing at one terminal is allowed for isomiR identification. 3 isomiRs should be within 18 to 26 nt in length. 4 isomiRs overlaps at least 16 nt with canonical isomiRs are identified as canonical isomiRs. 5 To investigate the sensitivity of them, isomiRs with only one read counts should be identified from the datasets.

In regard of accuracy, we investigate that based on four feature: 1 If the length of isomiR is beyond the interval we set. 2 If the expression of isomiR is counted inaccurately. 3 If the isomiR is contaminated with canonical miRNAs. 4 If the isomiR is attached to correct the pre-miRNA. Based on the validation, isomiR2Function showed great accuracy to all these features but canonical miRNAs are mixed in the identified isomiRs of isomiRID (Table 1). Additionally, there are several isomiRs which are longer than 26 nt in isomiRID results (Table 1). This analysis proved that isomiR2Function showed better accuracy than isomiRID.

Table 1 Accuracy investigation between isomiR2Function and isomiRID

	isomiR2Function						isomiR2ID					
	Templated			Non-templated			Templated			Non-templated		
	ath	bdi	osa	ath	bdi	osa	ath	bdi	osa	ath	bdi	osa
Too long	0	0	0	0	0	0	0	4	3	31	5	100
Too short	0	0	0	0	0	0	0	0	0	0	0	0
Wrong read count	0	0	0	0	0	0	0	0	0	0	0	0
Canonical miRNA	0	0	0	0	0	0	79	167	198	0	0	0
Wrong annotation	0	0	0	0	0	0	0	0	0	0	0	0

In addition to isomiR identification, graphic alignments of isomiRID was not accurate too (Figure 1). In the example bellow, isomiR 'TTCGGACCAGGCTTCATTTTTTTT' was wrongly aligned in the isomiRID result but not in isomiR2Function result (Figure 1). It was said the edited bases would be in lower case by isomiRID manual. Here, only the last base was in lower case instead of all edited bases in isomiRID result. However, the edited bases were showed in the exactly lower case in isomiR2Function one (Figure 1). Besides ath-miR166a-5p and ath-miR166a-3p, canonical miRNAs identified from other precursor were aligned to ath-MIR166a which might cause confusion in isomiRID result. However, in isomiR2Function one, only ath-miR166a-5p and ath-miR166a-3p were aligned to ath-MIR166a.

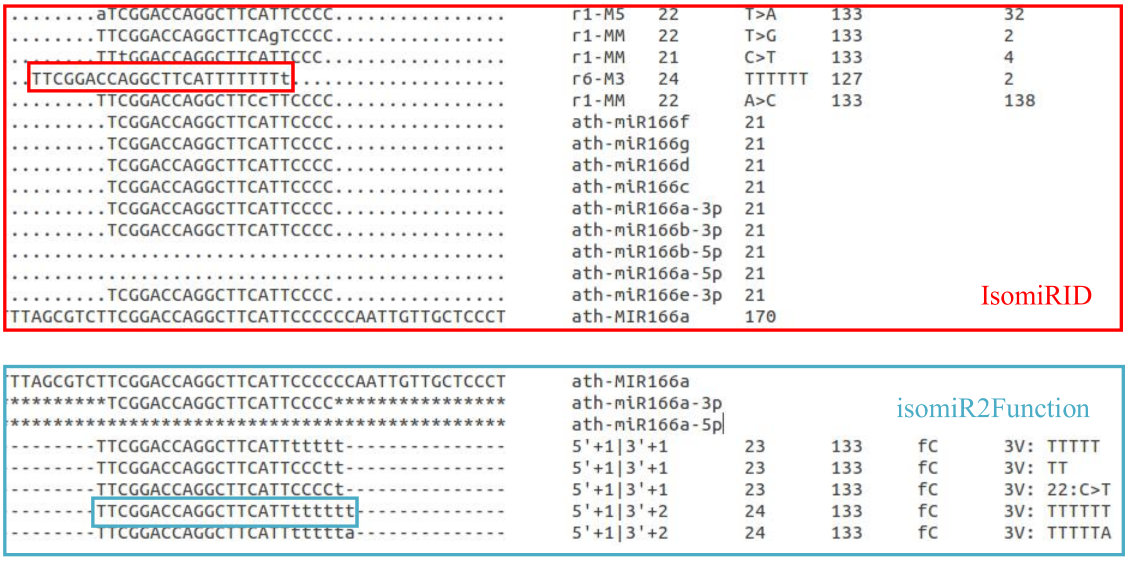

Figure 1 Graphic alignments of isomiRID and isomiR2Function Screenshot of

isomiRs aligned to ath-MIR166a. The same isomiR showed different alignment was highlighted in both isomiR2Function and isomiRID result.

In regard of sensitivity, isomiR2Function and isomiRID showed similar sensitivity for templated isomiR identification (Table 2). However, for non-templated ones, lots of them were missed in isomiRID results (Table 2). All those expected isomiRs were identified by isomiR2Function, which means isomiR2Function showed better sensitivity against isomiRID. All inputs, outputs and scripts used here for comparison are uploaded at https://drive.google.com/open?id=0B-w2z1YjW6TieG9jTUxhSmNwTTQ within file comparison.tar.bz2. In each folder the commands used for comparison are in the comment.sh and Script.R. All results can be repeated using those commands there.

Table 2 Sensitivity investigation between isomiR2Function and isomiRID

	isomiR2Function						isomiRID					
	templated			non-templated			templated			non-templated		
	ath	bdi	osa	ath	bdi	osa	ath	bdi	osa	ath	bdi	osa
Total	383	1613	2734	3959	8881	10946	383	1613	2734	3905	6869	10946
Complex	120	230	541	2299	2471	3836	120	230	541	2284	2270	3836
Canonical	200	629	836	1504	3748	2921	200	629	836	1499	3108	2921
Non-canonica	63	754	1357	156	2662	4189	63	754	1357	122	1491	4189
5a	14	28	33	250	133	82	14	28	33	250	111	82
3a	41	42	61	392	491	299	41	42	61	391	474	299
5d	17	53	89	29	440	189	17	53	89	29	411	189
3d	47	98	132	149	553	323	47	98	132	148	520	323
5a3a	6	9	18	69	39	30	6	9	18	67	22	30
5a3d	50	164	219	212	461	442	50	164	219	212	242	442
5d3a	17	210	224	44	511	549	17	210	224	44	289	549
5d3d	8	25	60	8	97	67	8	25	60	7	62	67
No drift	0	0	0	351	1023	940	0	0	0	351	977	940
Both	383	1613	2734	3905	6869	7877	383	1613	2734	3905	6869	7877
Specific	0	0	0	54	2012	3069	0	0	0	0	0	0

ADVANTAGES

As compared to the previously developed workflows, isomiR2Function allows for the following enhanced functionalities: 1. It allows for customizable length range, seed corrupt tolerance and minimum sequencing depth for isomiR identification; 2. Identification of isomiR on pre-miRNA not overlapping with canonical miRNAs; 3. Information on isomiR biogenesis using Ks plots; 4. Support for differential expression analysis of the identified isomiRs using DESeq and EBSeq; 5. Detailed classification of multiple internal substitution. isomiR2Function classifies isomiRs with multiple internal substitution in more detail, *i.e.* MS for having internal random SNPs, CV for having both internal SNPs and terminal substitutions and TS for having internal tandem SNPs; 6. Support for transcript based target identification using TargetFinder or degradome based PARE target identification using Cleaveland; 7. Functional enrichment of the isomiR targets for biological implications. Taking into account the implemented functionalities, we believe that isomiR2Functions will facilitate the large scale discovery of isomiRs across the plant species to gain and strengthen the role of the miRNA variants in post-transcriptional regulatory events.
